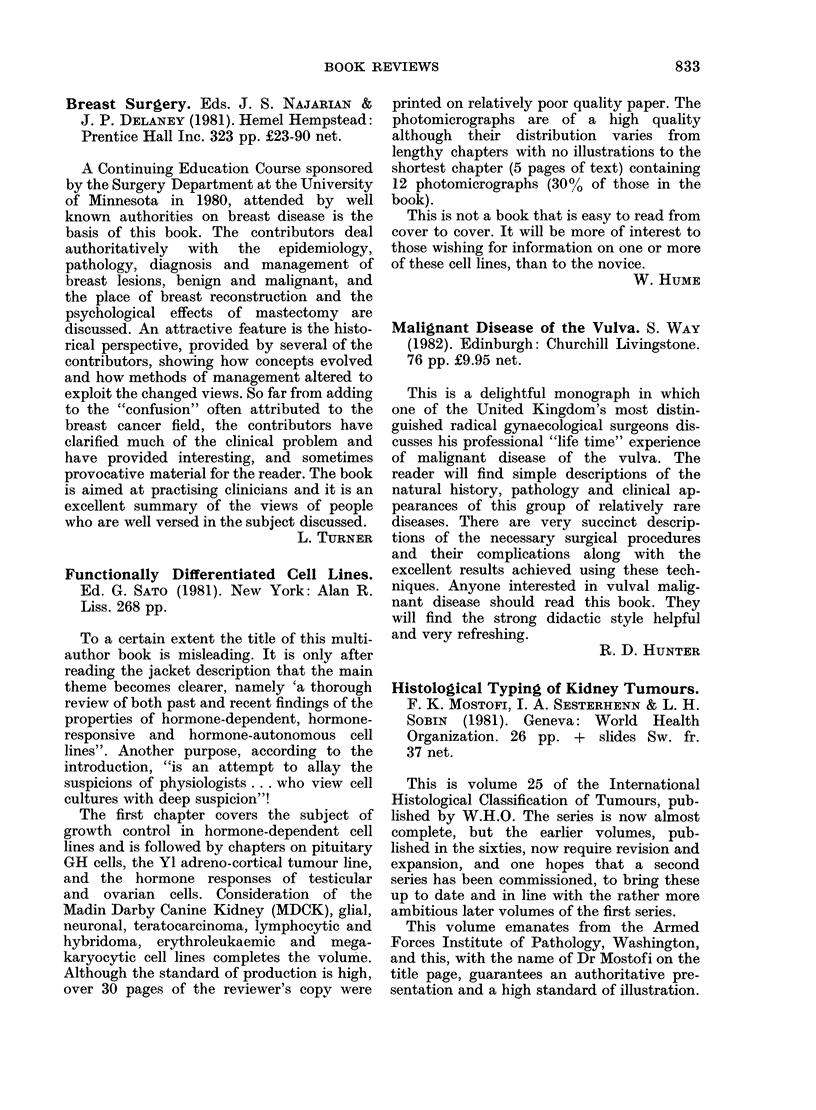# Breast Surgery

**Published:** 1982-11

**Authors:** L. Turner


**Breast Surgery.** Eds. J. S. NAJARIAN & J. P. DELANEY (1981). Hemel Hempstead: Prentice Hall Inc. 323 pp. £23-90 net.

A Continuing Education Course sponsored by the Surgery Department at the University of Minnesota in 1980, attended by well known authorities on breast disease is the basis of this book. The contributors deal authoritatively with the epidemiology, pathology, diagnosis and management of breast lesions, benign and malignant, and the place of breast reconstruction and the psychological effects of mastectomy are discussed. An attractive feature is the historical perspective, provided by several of the contributors, showing how concepts evolved and how methods of management altered to exploit the changed views. So far from adding to the "confusion" often attributed to the breast cancer field, the contributors have clarified much of the clinical problem and have provided interesting, and sometimes provocative material for the reader. The book is aimed at practising clinicians and it is an excellent summary of the views of people who are well versed in the subject discussed.

L. TURNER